# Confirmation of the Unidimensionality of the Satisfaction and Recovery Index Among Those With Various Musculoskeletal Disorders

**DOI:** 10.7759/cureus.62501

**Published:** 2024-06-16

**Authors:** Hiroshi Takasaki, Soma Ishida

**Affiliations:** 1 Department of Physical Therapy, Saitama Prefectural University, Koshigaya, JPN

**Keywords:** survey, reliability and validity, patient reported outcome measures, musculoskeletal disorders, factor analysis

## Abstract

Background

A semistructured patient-reported outcome measure (PROM) wherein patients rate the importance of structured items and the magnitude of the psychometric properties to be investigated (e.g., disability and satisfaction) facilitates patient engagement in their treatment and patient-centered clinical practice. The Satisfaction and Recovery Index (SRI) is one such semistructured PROM that was originally developed to measure recovery from a whiplash injury. Exploratory factor analysis demonstrated a one-factor structure among ambulatory community-dwelling people with traumatic musculoskeletal injuries. However, a confirmatory factor analysis has not been conducted among patients with various musculoskeletal disorders, and the internal structure of the SRI has not been established yet. Thus, this study aimed to investigate the internal structure of the SRI among patients with diverse musculoskeletal disorders.

Methodology

An anonymous survey was performed for patients who were referred for physical therapy for musculoskeletal disorders at a local orthopedic clinic. A confirmatory factor analysis was conducted. The goodness-of-fit criteria were as follows: chi-square/degree of freedom < 3, goodness-of-fit index > 0.90, adjusted goodness-of-fit index > 0.95, and root mean square error of approximation < 0.08.

Results

Data from 217 participants were analyzed. All goodness-of-fit criteria were satisfied.

Conclusion

This study confirmed the acceptable internal structure of the SRI among patients with diverse musculoskeletal disorders.

## Introduction

In patient-centered clinical practice, the subjective status of the patient must be understood, and a patient-reported outcome measure (PROM) is needed. The integration of PROM facilitates patient engagement in their treatment [1]. A semistructured PROM wherein patients rate the importance of structured items and the magnitude of the psychometric properties to be investigated (e.g., disability and satisfaction) is considered a feasible and promising patient-centered PROM [2], and some have been developed recently [3-5]. Among these semistructured PROMs, the Satisfaction and Recovery Index (SRI) is a potentially applicable region-agnostic PROM [4,6].

Originally, the SRI was developed to measure recovery from a whiplash injury via a focus group of patients with whiplash injuries, and cognitive interviews with patients with upper extremity disorders were conducted [4]. In the SRI, patients rate the importance of nine items and their satisfaction on two 11-point scales. Among ambulatory community-dwelling people with traumatic musculoskeletal injuries, the SRI demonstrated single-factor loading in the exploratory factor analysis, convergent validity with the SF-12v2® Health Survey [7], and better responsiveness than the SF-12v2® Health Survey [4]. However, the unidimensionality of the SRI has not been established as a confirmatory factor analysis has not been performed yet. Furthermore, during the development of a Japanese version, patients with various musculoskeletal disorders were found to be able to understand the SRI [6].

Therefore, this study aimed to investigate the internal structure of the SRI among patients with diverse musculoskeletal disorders. The unidimensionality of the SRI was hypothesized to be acceptable in those patients.

## Materials and methods

Design

Because an anonymous survey was performed, the need for informed consent was waived. This study was approved by the Ethics Committee of the Saitama Prefectural University (No. 22040).

Participants

Convenience sampling was used to recruit participants from June 2021 to May 2024. Inclusion criteria were (1) patients who were referred for physical therapy for musculoskeletal disorders at a local orthopedic clinic (Tokyo, Japan), (2) ≥ 18 years old, and (3) whose first language is Japanese. Patients with respiratory, neurological, or cognitive comorbidities (e.g., dementia) who were diagnosed by a medical doctor were not eligible. Participants were recruited until 200 analyzable SRI data were collected, considering the adequate quality criterion for the examination of construct validity according to the COnsensus-based Standards for the selection of health Measurement INstruments (COSMIN) guidelines [8,9].

Variables

Before the initial physical therapy session, participants were asked to respond to the survey. This study collected data including age, sex, duration of symptoms that resulted in a referral for physical therapy (≤ 7 days, eight days to three months, and ≥ three months), location of the symptoms, and pain intensity using the four-item pain intensity measure (P4) and the SRI. The duration of symptoms was defined as the period since the last time the patient had no such symptoms at all for > 1 month [10-12]. The symptom location was assessed using a body chart with 23 areas [13-15]. The P4 is a valid and reliable PROM for subjective pain intensity on a four-item 11-point numerical rating scale to indicate the average pain intensity over the previous two days in the morning, afternoon, and evening and during activities [16,17]; the higher the total P4 score, the greater the perceived pain intensity (0-40).

The SRI is composed of 10 items, where one item (item 6) is a dummy to check whether the respondents fill a number mindlessly. The SRI score is calculated based on the following two steps from nine items: (1) calculating a weighted score for each item and (2) calculating the total score.

Analysis

A confirmatory factor analysis was conducted with Analysis of Moment Structures Statistical Product and Service Solutions (AMOS SPSS) (version 20; IBM SPSS Statistics for Windows, Armonk, NY) for the weighted scores of the nine items. The goodness-of-fit criteria were as follows: chi-square/degree of freedom < 2 (good fit) and < 3 (acceptable fit), goodness-of-fit index > 0.95 (good fit) and > 0.90 (acceptable fit), adjusted goodness-of-fit index > 0.97 (good fit) and > 0.95 (acceptable fit), and root mean square error of approximation < 0.05 (good fit) and < 0.08 (acceptable fit) [18,19]. Data with missing SRI values or dummy items with inappropriate responses were excluded from the analysis. A post hoc model modification was conducted according to the suggested modification indices.

Cronbach’s alpha was calculated for internal consistency. The values were interpreted as acceptable (≥ 0.7) and not acceptable (< 0.7) [9]. In the SRI, the total score is calculated by weighting item importance. Therefore, the item-total correlation (ITC) was assessed using Pearson’s correlation coefficient (r). The correlation between the weighted score of each item and the total SRI score excluding the item was calculated. Values of ≤ 0.2 were interpreted as acceptable and > 0.2 as not acceptable [20,21].

## Results

**Figure 1:**
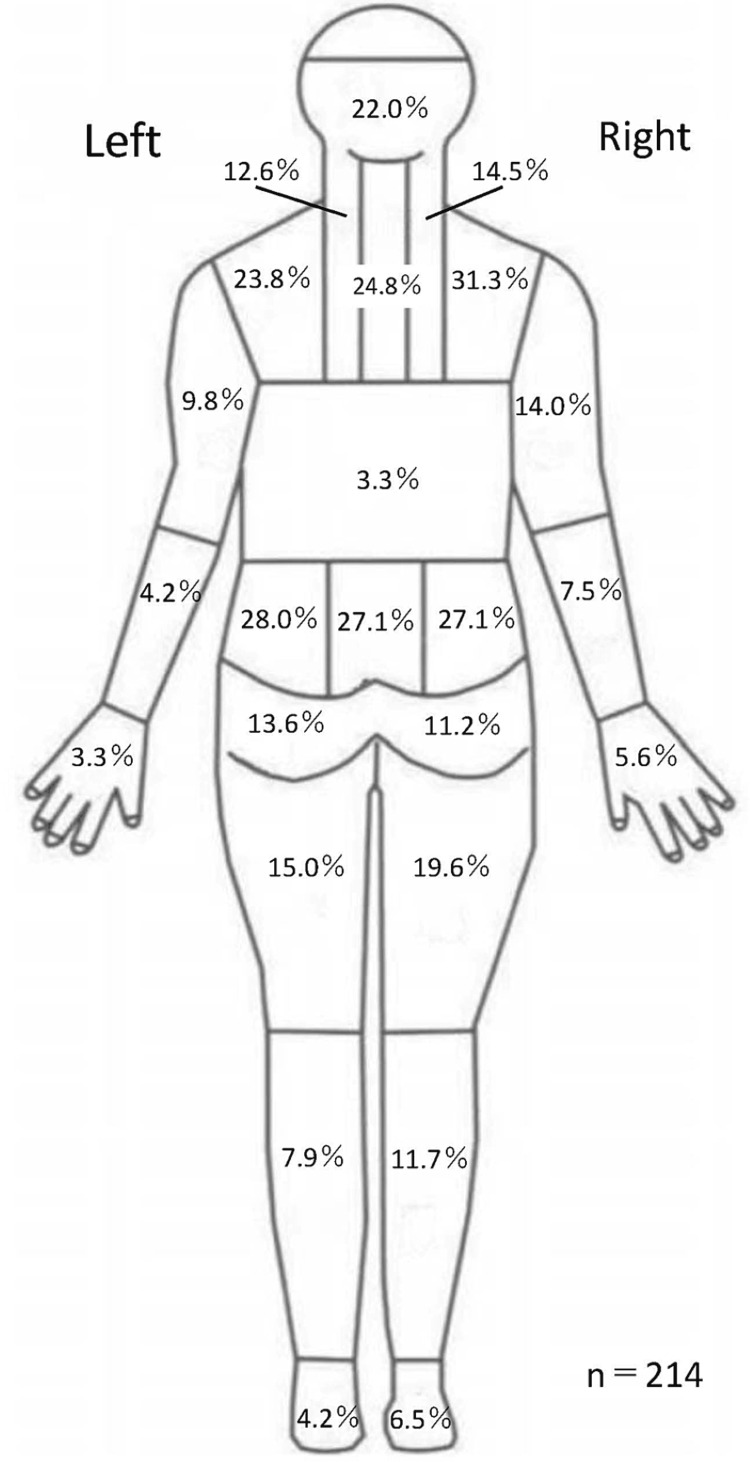
Symptom distributions of the 217 participants.

Approximately 233 patients participated in the study. There were 16 participants who had missing data on SRI and were excluded, and data from 217 participants were analyzed (Table 1). The proportion of symptom locations is shown in Figure 1.

**Table 1:** Summary of the 217 participants. Abbreviation; SD, standard deviation; n, number. ^*^n = 215; †n = 213; ​​​​​​​^‡^n = 216

Variables	N = 217
Age^*^ (years), mean (SD)	48.5 (17.8)
Sex^†^	
n of males (%)	98 (46%)
n of females (%)	115 (54%)
Symptom duration^‡^	
n of those with < 7 days (%)	16 (7%)
n of those with 8 days–3 months (%)	116 (54%)
n of those with > 3 months (%)	84 (39%)
4-item Pain Intensity Measure^‡^ (0–40), mean (SD)	14.9 (8.7)
Satisfaction and Recovery Index score (0–100), mean (SD)	73.7 (15.2)

**Figure 2:**
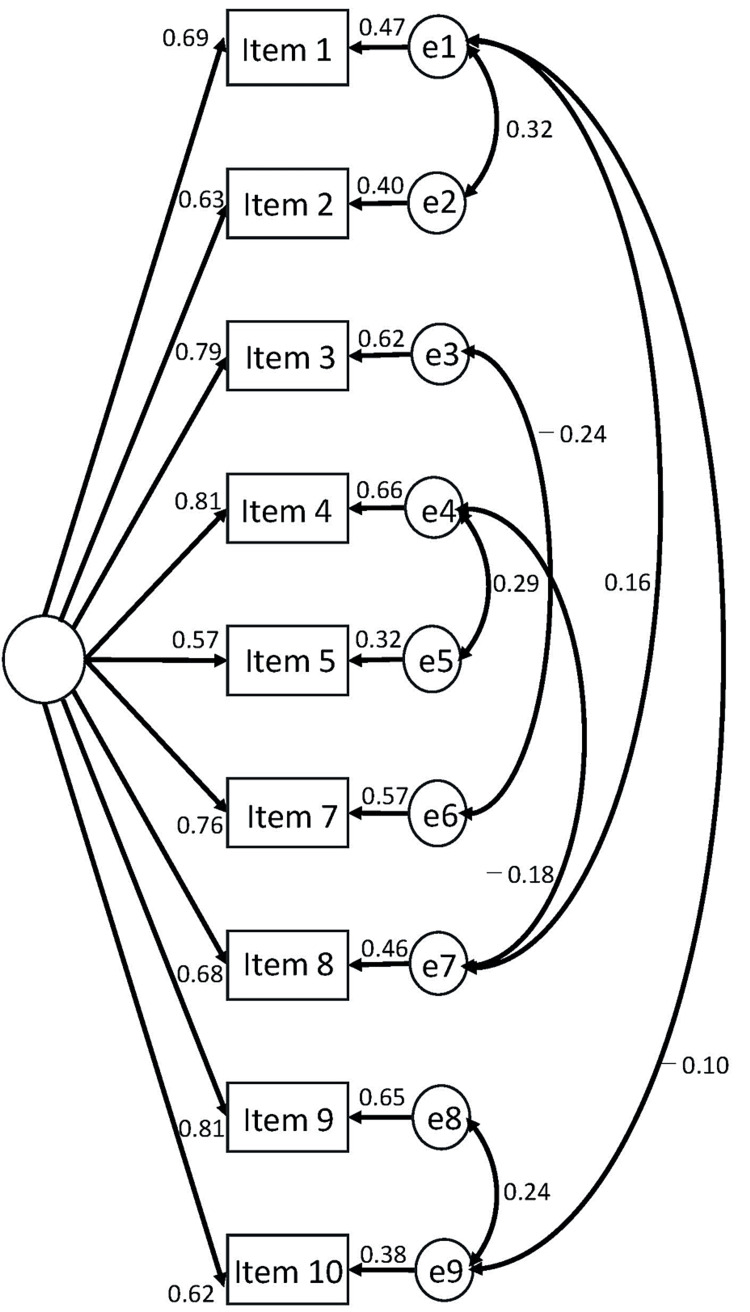
The structure model with standardized coefficients.

Figure 2 presents the structure of the models. Table 2 shows the goodness-of-fit criteria, satisfying all criteria of good or acceptable fit.

**Table 2:** Goodness-of-fit results. Chi-square/degree of freedom < 2 (good fit) and < 3 (acceptable fit); goodness-of-fit index > 0.95 (good fit) and > 0.90 (acceptable fit); adjusted goodness-of-fit index > 0.97 (good fit) and > 0.95 (acceptable fit); root mean square error of approximation < 0.05 (good fit) and < 0.08 (acceptable fit).

Goodness-of-fit statistics	Value
Chi-square/degree of freedom	1.051
Goodness-of-fit index	0.980
Adjusted goodness-of-fit index	0.954
Root mean square error of approximation	0.015

Acceptable internal consistency was confirmed with a Cronbach’s alpha of 0.902 (95% confidence intervals: 0.881-0.920). ITCs are presented in Table 3, where all items satisfied the predetermined criteria.

**Table 3:** Item-total correlation with Pearson’s correlation coefficient.

Item No.	Pearson’s R	P value	95% confidence intervals
1	0.68	< 0.001	0.60–0.75
2	0.60	< 0.001	0.51–0.68
3	0.66	< 0.001	0.58–0.73
4	0.70	< 0.001	0.62–0.76
5	0.51	< 0.001	0.40–0.60
7	0.60	< 0.001	0.50–0.68
8	0.62	< 0.001	0.53–0.70
9	0.73	< 0.001	0.66–0.79
10	0.57	< 0.001	0.47–0.65

## Discussion

In this study, a confirmatory factor analysis demonstrated that all items met the goodness-of-fit criteria of being either good or acceptable. Therefore, this result suggests that the SRI exhibits a single-factor structure among patients with diverse musculoskeletal diseases. The internal consistency was also acceptable. These findings indicate that the second step of the reliability and validity testing process indicated in the COSMIN (i.e., the confirmation of the internal structure, has been completed). In the future, the third step of the reliability and validity testing process as indicated in COSMIN (i.e., test-retest reliability and responsiveness) must be verified. Particularly, clinical trials are recommended to use the method of determining whether the 95% confidence interval of the difference exceeds the minimum clinically important difference (MCID), rather than a statistically significant difference [22]. Such a new clinical trial has begun to be reported [12]. Therefore, the verification of the MCID of the SRI is considered a priority research agenda.

The total SRI score is divided by the total number of importance scores, which is an ingenious way to enhance the individuality of each patient. Herein, the ITC was evaluated to confirm the structural validity of the total score, even with this unique calculation method. Consequently, all items met the ITC criteria. Therefore, the internal structure of the total SRI score is deemed reliable.

Previous studies have commonly used pain intensity, degree of functional impairment, and cost-effectiveness as primary outcomes. However, patients’ demands for an intervention are diverse, and developing a new outcome that reflects patient individuality was one of the research priorities in musculoskeletal research [23]. However, the above concern, namely, that the intervention effects cannot be truly reflected, would arise because the value of outcomes such as pain intensity, functional limitations, and cost-effectiveness vary among patients. Therefore, evaluating the effectiveness of a patient-centered approach when the outcome is well-being, a psychological characteristic that is the goal for any patient [24], may be possible. Such a conceptually higher level of outcomes for well-being would include life satisfaction, which is measured by the SRI. To the best of the author's knowledge, SRI is the only free, semistructured PROM for life satisfaction. The SRI may be a promising primary outcome for future clinical trials of musculoskeletal disorders as this study confirmed that the SRI has sufficient internal structure in patients with various musculoskeletal disorders.

One limitation of this study is that data were collected in a single center. Second, because this was an anonymized survey and considering patients might not remember their exact diagnosis, we asked only for the symptomatic area rather than the name of the diagnosis. However, we do not believe that these limitations overshadow the results of this study. Nevertheless, there is a limitation that the present findings are based on the Japanese population, raising uncertainty regarding whether the SRI is unidimensional in other cultures. Verification of cross-cultural validity will be required in the future.

## Conclusions

This study confirmed the acceptable internal structure of the SRI among patients with diverse musculoskeletal disorders. Therefore, it is now possible to verify the third step of the reliability and validity testing process as indicated in COSMIN to use the SRI in clinical practice. The SRI is a semistructured and region-agnostic PROM that is free of charge and thus may be widely used to facilitate patient-centered interventions for patients with musculoskeletal disorders.
